# The *slc4a2b* gene is required for hair cell development in zebrafish

**DOI:** 10.18632/aging.103840

**Published:** 2020-10-12

**Authors:** Fuping Qian, Xin Wang, Zhenhua Yin, Gangcai Xie, Huijun Yuan, Dong Liu, Renjie Chai

**Affiliations:** 1 MOE Key Laboratory of Developmental Genes and Human Disease, School of Life Science and Technology, Jiangsu Province High-Tech Key Laboratory for Bio-Medical Research, Southeast University, Nanjing 210096, China; 2 School of Life Sciences, Key Laboratory of Neuroregeneration of Jiangsu and Ministry of Education, Co-innovation Center of Neuroregeneration, Nantong University, Nantong 226019, China; 3 Medical School, Nantong University, Nantong 226019, China; 4 Medical Genetics Center, Southwest Hospital, Army Medical University, Chongqing 400038, China; 5 Institute for Stem Cell and Regeneration, Chinese Academy of Sciences, Beijing, China; 6 Beijing Key Laboratory of Neural Regeneration and Repair, Capital Medical University, Beijing 100069, China

**Keywords:** slc4a2b gene, hair cell, neuromast, apoptosis, zebrafish

## Abstract

Hair cells (HCs) function as important sensory receptors that can detect movement in their immediate environment. HCs in the inner ear can sense acoustic signals, while in aquatic vertebrates HCs can also detect movements, vibrations, and pressure gradients in the surrounding water. Many genes are responsible for the development of HCs, and developmental defects in HCs can lead to hearing loss and other sensory dysfunctions. Here, we found that the *solute carrier family 4, member 2b* (*slc4a2b*) gene, which is a member of the anion-exchange family, is expressed in the otic vesicles and lateral line neuromasts in developing zebrafish embryos. An *in silico* analysis showed that the *slc4a2b* is evolutionarily conserved, and we found that loss of function of *slc4a2b* resulted in a decreased number of HCs in zebrafish neuromasts due to increased HC apoptosis. Taken together, we conclude that *slc4a2b* plays a critical role in the development of HCs in zebrafish.

## INTRODUCTION

Mechanosensory hair cells (HCs) in the inner ear play a crucial role in sensing auditory and vestibular signals in the environment, and HC damage is a leading cause of hearing loss. Congenital hearing loss occurs in approximately 1/500 infants, and genetic factors account for at least 50% of cases [1]. Thus heritable hearing loss caused by developmental defect of HCs due to mutations in deafness genes is a significant contributor to reduced quality of life. The functions of deafness genes are mainly involved in cochlear homeostasis, cellular organization, coding for tectorial membrane-associated proteins, neuronal transmission, and cell growth, differentiation, and survival. To date a total of 121 non-syndromic hearing loss genes have been identified (https://hereditaryhearingloss.org, most recent update: 4/20/2020); however, there are likely to be many deafness genes waiting to be discovered. Therefore, identifying deafness genes is crucial for protecting HCs from injury and thus for preventing and treating hereditary hearing loss.

Ion homeostasis is essential for maintaining cell survival and proper cell function, and many hearing disorders are caused by inner ear HC ion homeostatic disruption. Therefore, membrane-associated ion channels, transporters, and exchangers are required for HC function, and loss of function of these proteins induces HC loss and thus leads to hearing deficits.

The solute carrier (SLC) group of membrane transport proteins consists of more than 60 families and over 400 members. Mutations of some SLC genes have been shown to lead to hearing impairments or HC defects, for example, human *SLC44A4* [2] and zebrafish *slc26a2* [3]. The HCO_3_^−^/Cl^−^ anion exchangers (AEs) belong to the SLC4 family and include three primary members, namely AE1 (or SLC4A1), AE2 (or SLC4A2), and AE3 (or SLC4A3), each of which can exchange HCO_3_^−^ for Cl^−^ across the plasma membrane and thus play an important role in maintaining the intracellular pH (pHi), Cl^−^ concentrations, and cell volume [4]. Among the three genes, the *SLC4A2* gene appears to be more widely distributed than the other two, and it has been shown to be expressed in the inner and outer HCs and in the supporting cells of rat and guinea pig cochleae [5] and in the HCs, the supporting cells between the inner and outer sulcus cells, type I, II, III, and V fibrocytes of the spiral ligament, Reissner’s membrane cells, spiral limbus cells, and spiral ganglion neurons in the common marmoset cochlea [6]. *SLC4A2* is required for proper osteoclast differentiation and function in mice [7], and mutations in bovine *SLC4A2* are associated with osteopetrosis in Red Angus cattle [8]. SLC4A2-mediated HCO_3_^−^/Cl^−^ exchange activity is essential for calpain-dependent regulation of the actin cytoskeleton in osteoclasts [9], and SLC4A2 is also involved in pHi homeostasis [10], spermatogenesis [11], gastric acid secretion [12], and immune responses [13, 10]. However, the role of *SLC4A2* in hearing has not been explored.

In zebrafish, there are two unlinked genes that are equally closely related to the *SLC4A2* genes in mammals, namely *slc4a2a* (also called *ae2*, *ae2.1*, and *slc4a2*) and *slc4a2b* (also called *ae2.2*) [14, 15]. *slc4a2a* mRNA can be detected in the prospective midbrain as early as the five-somite stage, then later in the pronephric primordia and in the developing pronephric duct, where it persists through 72 hpf (hours post fertilization), and it mediates both Na-independent electroneutral Cl^−^/Cl^−^ exchange and Cl^−^/HCO_3_^−^ exchange [14]. We demonstrate here that the *slc4a2b* gene is expressed in the otic vesicle and lateral line neuromasts in zebrafish. An *in silico* analysis showed that the *slc4a2b* gene is highly evolutionarily conserved, and we further show that loss of function of *slc4a2b* resulted in decreased numbers of posterior lateral line neuromasts, HC clusters, and HCs due to increased HC apoptosis. Taken together, our findings highlight a critical role for the *slc4a2b* gene in HC development, and thus *slc4a2b* might be a newly identified deafness-related gene.

## RESULTS

### The *slc4a2b* gene is evolutionarily conserved and is expressed in the otic vesicle and the lateral line neuromasts in zebrafish

**Figure 1:**
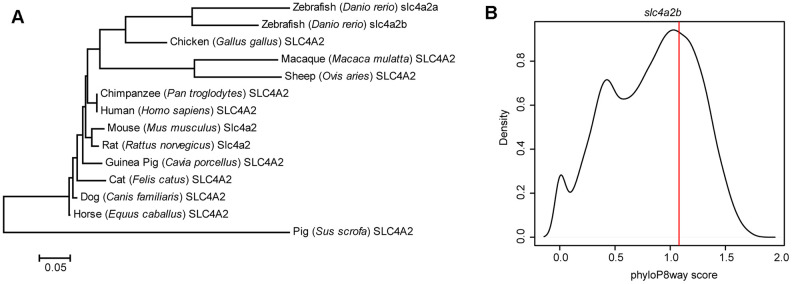
**Evolutionary feature analysis of the *slc4a2b* gene.** (**A**) Evolutionary relationships of SLC4A2 in different species. The evolutionary history was inferred using the Neighbor-Joining method, and the optimal tree with the sum of branch length = 1.66619414 is shown. The tree is drawn to scale, with branch lengths in the same units as those of the evolutionary distances used to infer the phylogenetic tree. The evolutionary distances were computed using the Poisson correction method and are in the units of the number of amino acid substitutions per site. The analysis involved 14 amino acid sequences. All positions containing gaps and missing data were eliminated. There were a total of 237 positions in the final dataset. The evolutionary analysis was conducted in MEGA6. (**B**) *slc4a2b* phyloP8way conservation score (vertical red line) and the zebrafish protein coding gene phyloP8way conservation score density distribution (black line). The x-axis is the average phyloP8way score for the coding sequence region of the gene, and the y-axis is the density of the number of genes in each conservation score range.

For a better understanding of the *slc4a2b* gene in zebrafish, we first analyzed the evolutionary features of the gene. As shown in Figure 1A, the zebrafish *slc4a2b* gene has significant amino acid sequence similarities to other species. Moreover, the phyloP8way conservation score of *slc4a2b* is greater than more than half of the zebrafish protein-coding genes (Figure 1B), and is ranked 7,365 among over 23,000 genes in zebrafish, which indicates its evolutionary conservation.

**Figure 2:**
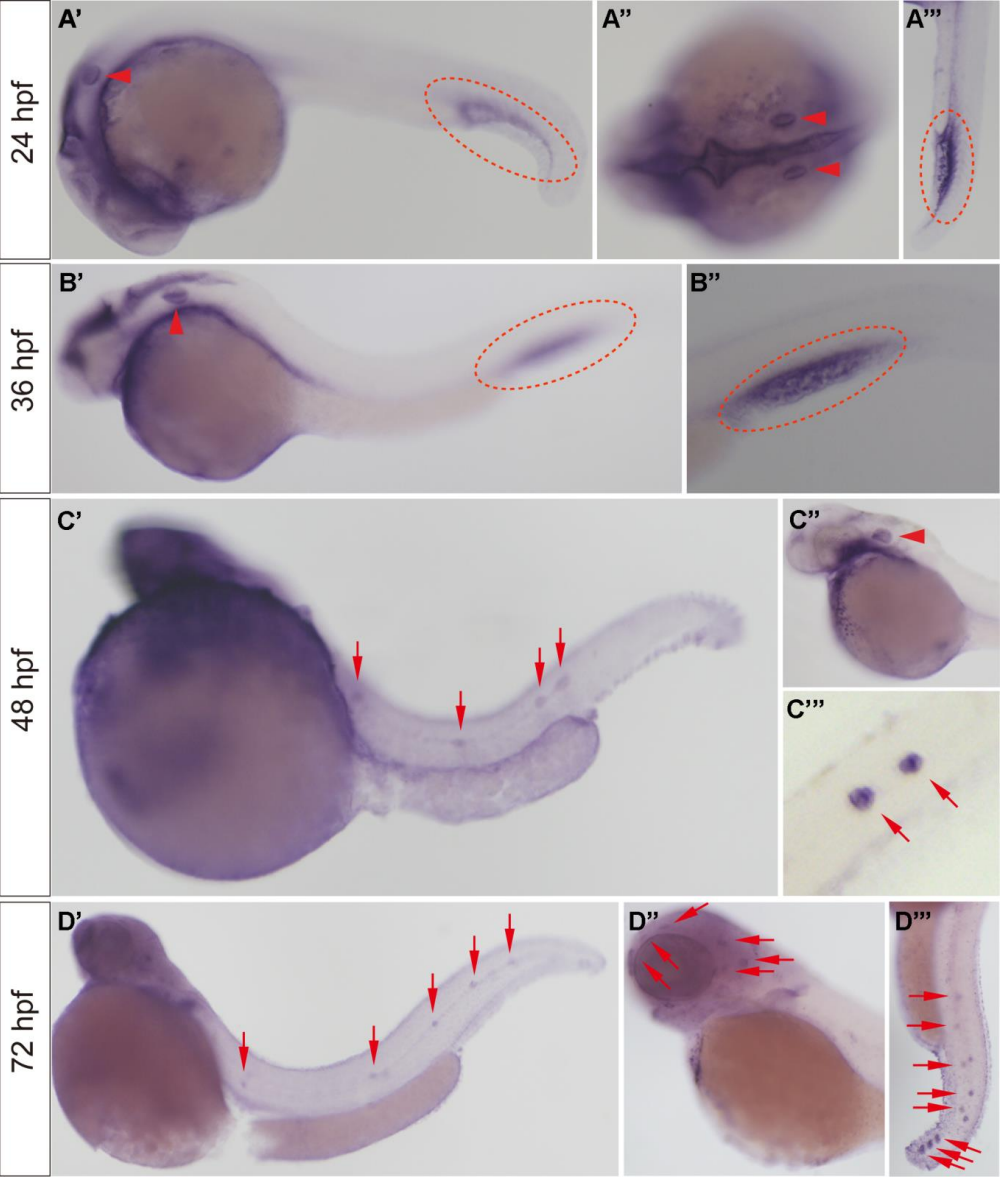
***slc4a2b* mRNA expression pattern detected by WISH.** (**A**, **B**) The *slc4a2b* mRNA was mainly expressed in the otic vesicle (indicated by red arrowheads) and caudal vein (indicated by red dotted lines) at 24 hpf and 36 hpf. (**A**’’) shows the dorsal view of the otic vesicle, and (**A**’’’) and (**B**’’) are focused on the caudal vein. (**C**) At 48 hpf, *slc4a2b* mRNA was detected not only in the otic vesicle (indicated by the red arrowhead), but also in the lateral line neuromasts (indicated by red arrows). (**C**’’) and (**C**’’’) show the otic vesicle and the neuromasts at higher magnification, respectively. (**D**) *slc4a2b* mRNA was expressed in neuromasts at 72 hpf. (**D**’’) shows the neuromasts on the head (red arrowheads indicate the neuromast MI1, MI2, O2, et al.) and (**D**’’’) shows the posterior lateral line neuromasts.

WISH was used to detect the expression of the *slc4a2b* gene in developing zebrafish embryos. As shown in Figure 2, *slc4a2b* mRNA was observed in the otic vesicle and caudal vein at 24 and 36 hpf (Figure 2A, 2B), while at 48 hpf the *slc4a2b* gene was not only expressed in otic vesicle, but also in posterior lateral line neuromasts (Figure 2C). With continued growth, the *slc4a2b* mRNA was found in the neuromasts on the head (Figure 2D). Together these results show that the *slc4a2b* gene is highly conserved and is expressed in lateral line neuromasts in zebrafish, which suggest its important role in neuromast development.

### Knockdown of the *slc4a2b* gene leads to decreased neuromasts and HCs

**Figure 3:**
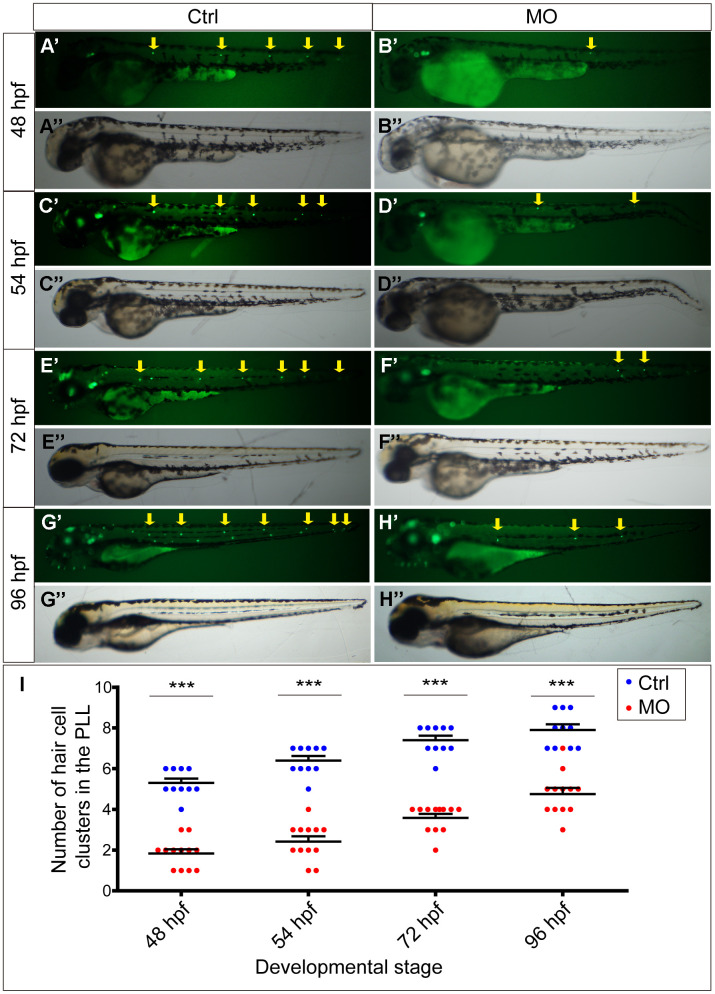
***slc4a2b* knockdown leads to decreased HC clusters in the posterior lateral line of zebrafish.** (**A**–**D**) Zebrafish injected with *slc4a2b*-morpholino (MO) had normal morphology (**B**’’, **D**’’, **F**’’, **H**’’) but decreased HC clusters in the posterior lateral line (**B**’, **D**’, **F**’, **H**’) compared to the controls (Ctrl) (**A**’’, **C**’’, **E**’’, **G**’’ and **A**’, **C**’, **E**’, **G**’) at different developmental stages. The HC clusters in the posterior lateral line are indicated by yellow arrows. (**I**) Quantification of the number of HC clusters in the posterior lateral line in *slc4a2b*-morphants and controls at different developmental stages. ***P < 0.001.

To determine the function of the *slc4a2b* gene, morpholino-mediated gene knockdown was used to downregulate its expression in the transgenic zebrafish line *Tg(Brn3c:mGFP)* [16] in which HCs are labeled by GFP. The *slc4a2b*-specific morpholino-injected larvae (Figure 3B’’, 3D’’, 3F’’, 3H’’) were morphologically similar at different stages to larvae injected with control morpholino (Figure 3A’’, 3C’’, 3E’’, 3G’’). However, fluorescence microscopic analysis showed that the HC clusters in the posterior lateral line of *slc4a2b* morphants (Figure 3B’, 3D’, 3F’, 3H’, 3I) decreased significantly at different stages in comparison to control larvae (Figure 3A’, 3C’, 3E’, 3G’, 3I).

**Figure 4:**
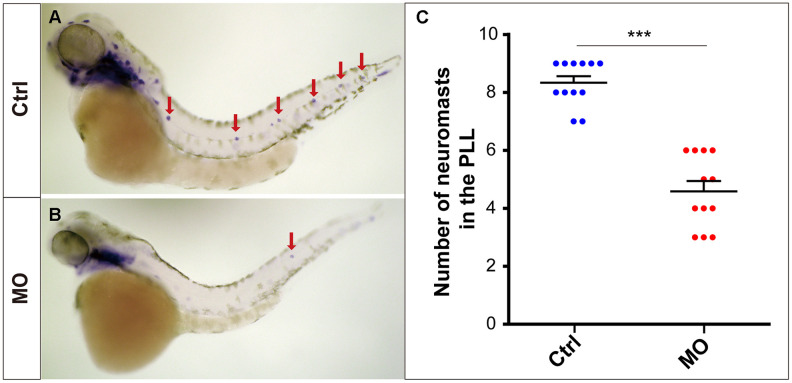
***slc4a2b* knockdown leads to decreased lateral line neuromasts in zebrafish.** Zebrafish larvae at 72 hpf and the *eya1* RNA probe were used in the WISH analysis. (**A**, **B**) *slc4a2b*-morphants (MO) (**B**) had fewer neuromasts (indicated by red arrows) compared to the controls (**A**). (**C**) Quantification of the number of the posterior lateral line neuromasts in *slc4a2b*-morphants and controls. ***P < 0.001.

The *eya1* gene is a marker of lateral line neuromasts in zebrafish [17, 18], and the *eya1*-specific WISH experiment provided powerful evidence that *slc4a2b* gene knockdown led to reduced numbers of neuromasts in zebrafish (Figure 4).

**Figure 5:**
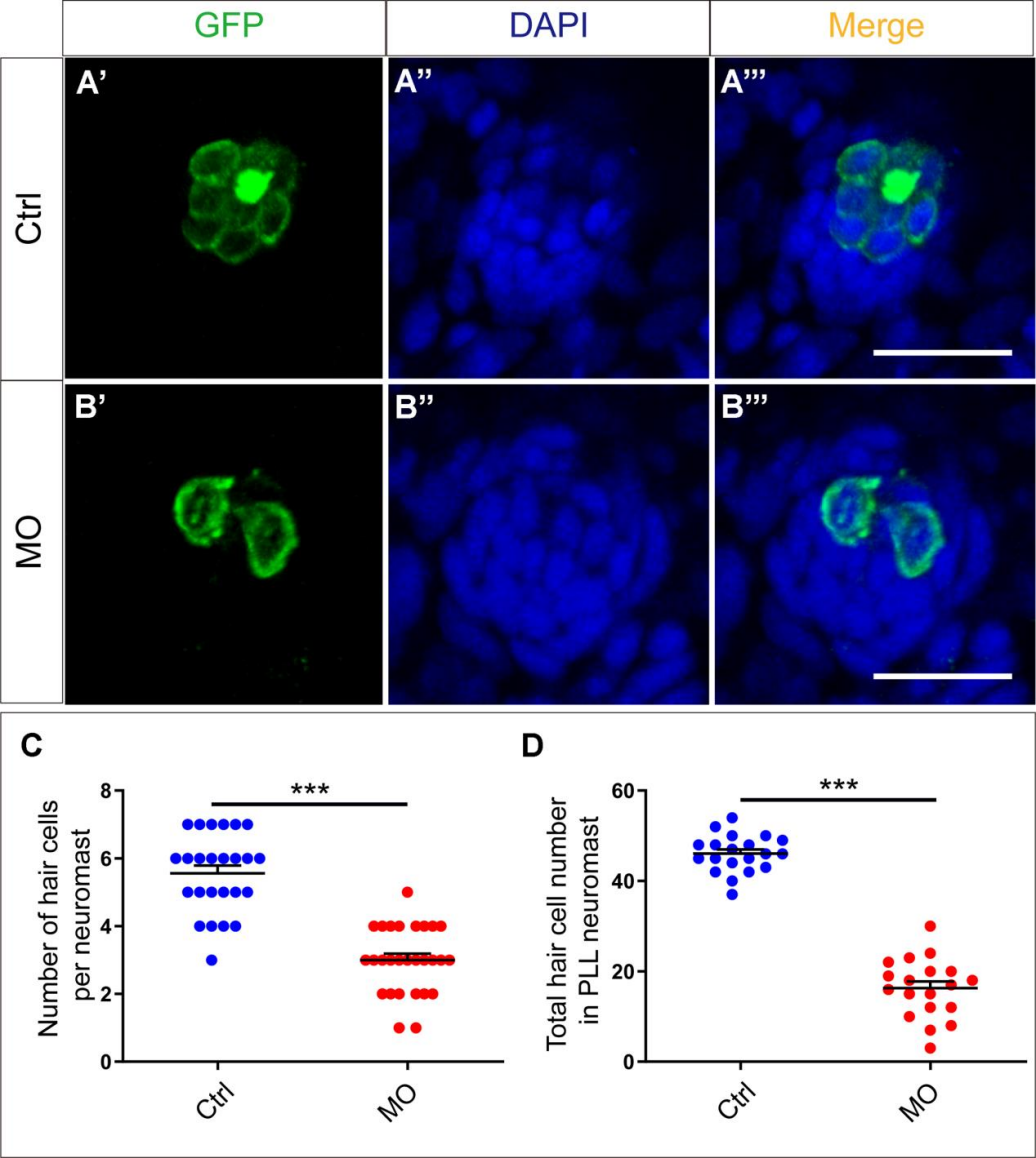
***slc4a2b* knockdown leads to decreased HCs in the posterior lateral line neuromast of zebrafish.** (**A**, **B**) *slc4a2b*-morphants (MO) (**B**) had fewer HCs in each neuromast compared to controls (**A**). *Tg(Brn3c:mGFP)* transgenic zebrafish were used in the analysis, and the cells labeled by GFP represent HCs, while DAPI was used to stain the cell nuclei. (**C**, **D**) Quantification of the number of HCs in each neuromast and the total HCs in posterior lateral line neuromasts of the *slc4a2b*-morphants and controls. Scale bar = 20 μm, *** *P* < 0.001).

Although *slc4a2b* gene knockdown led to decreased neuromasts and HC clusters, whether HCs in the remaining neuromasts were affected was not clear. To answer this, immunofluorescence experiments and confocal microscopic analysis were used to visualize the HCs. The HC number in the remaining neuromasts of the *slc4a2b* morphants was also reduced dramatically compared to controls (Figure 5A–5C), which further decreased the total HC number (Figure 5D). Taken together, these results suggest that *slc4a2b* gene knockdown has a negative effect on HC development in zebrafish.

### CRISPR/Cas9-mediated *slc4a2b* gene mutation disrupts HC development

**Figure 6:**
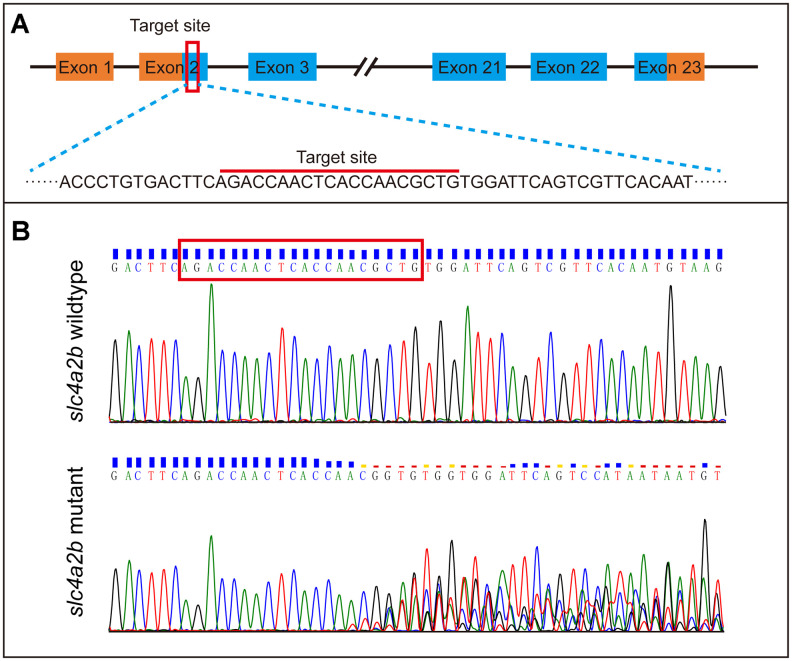
**Generation of *slc4a2b* gene mutant zebrafish using CRISPR/Cas9 gene-editing technology.** (**A**) The coding sequence in exon 2 of the *slc4a2b* gene was chosen to be the target of mutation. (**B**) Various mutations occurred in the target site of the *slc4a2b* gene in mutant zebrafish compared to the wild-type fish.

To further identify the role of the *slc4a2b* gene in HC development, we generated *slc4a2b* mutant zebrafish (namely, *Tg(Brn3c:mGFP);slc4a2b*^mut^) using CRISPR/Cas9 gene editing technology with *Tg(Brn3c:mGFP)* transgenic zebrafish. As shown in Figure 6A, an sgRNA targeting the coding sequence in exon 2 of the *slc4a2b* gene was synthesized *in vitro*. After co-injection of *slc4a2b*-specific sgRNA and Cas9 mRNA, the zebrafish were grown and the genomic DNA was extracted for genotyping. The mutations were observed at the target site within the genome (Figure 6B).

**Figure 7:**
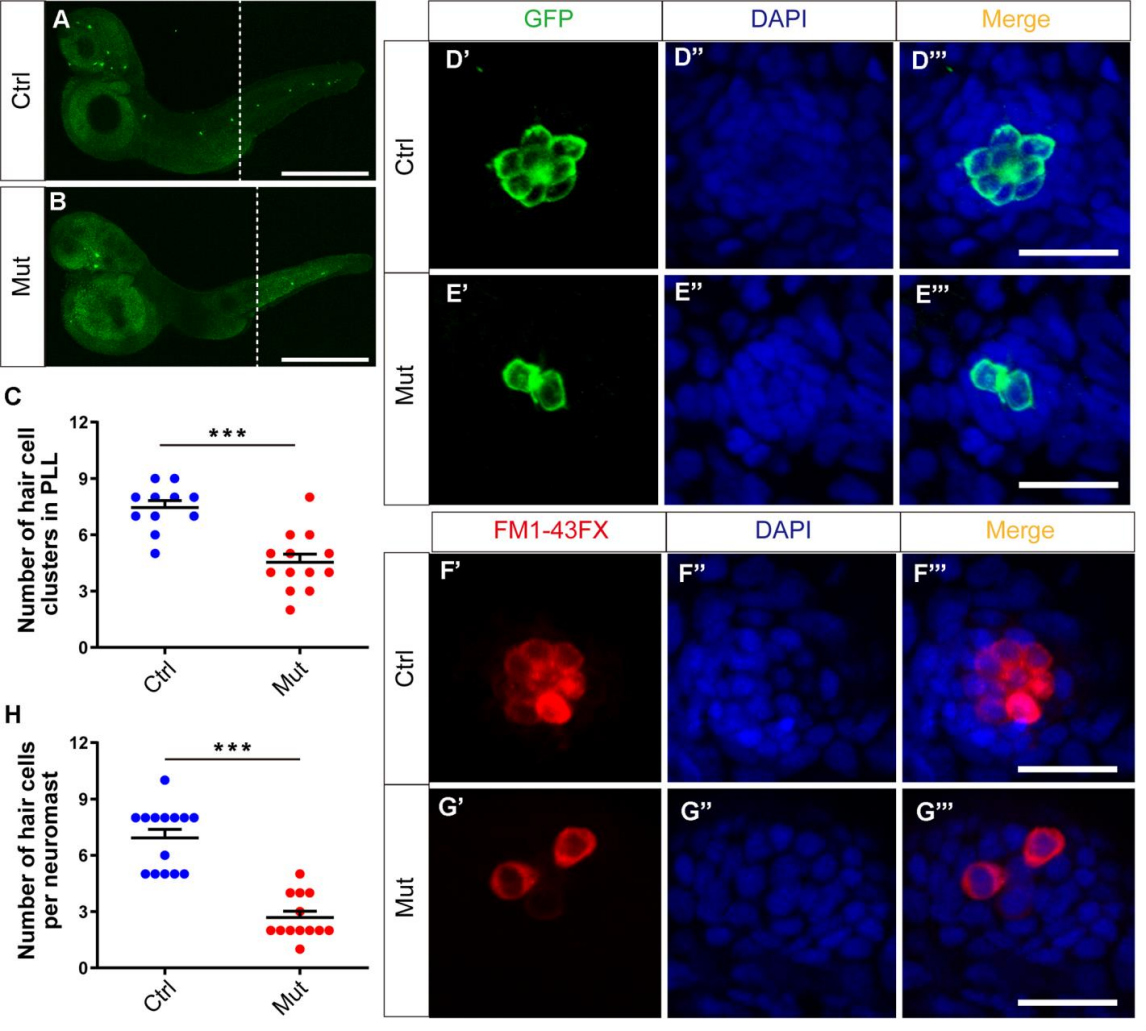
***slc4a2b* gene mutant zebrafish have fewer HC clusters and HCs in the posterior lateral line (PLL).** (**A**, **B**) The *Tg(Brn3c:mGFP);slc4a2b*^mut^ zebrafish had fewer HC clusters in the posterior lateral line compared to the controls. Here, the anti-GFP antibody was used to label the HCs. Scale bar = 500 μm. (**C**) Quantification of the number of HC clusters in the posterior lateral line of the *slc4a2b* gene mutant zebrafish and controls. ***P < 0.001. (**D**, **E**) The *Tg(Brn3c:mGFP);slc4a2b*^mut^ zebrafish had fewer HCs in each posterior lateral line neuromast compared to controls. Here, anti-GFP antibody and DAPI were used to label the HCs and the nuclei, respectively. Scale bar = 20 μm. (**F**, **G**) The *Tg(Brn3c:mGFP);slc4a2b*^mut^ zebrafish had fewer functional HCs in each posterior lateral line neuromast compared to controls. The successful staining with FM1-43FX dye was as a marker of functional HCs. Scale bar = 20 μm. (**H**) Quantification of the number of HCs in each neuromast of the *slc4a2b* mutant zebrafish and controls. *** *P* < 0.001.

The phenotype caused by *slc4a2b* gene defects was analyzed at 3 dpf. First, by immunostaining we found that the number of HC clusters in the posterior lateral line of *Tg(Brn3c:mGFP);slc4a2b*^mut^ zebrafish was decreased significantly compared to controls, which was similar to what was seen in the *slc4a2b* morphants (Figure 7A–7C). Here, an anti-GFP antibody was used to label the HCs. We further analyzed HC development in the neuromasts of the *slc4a2b* mutant zebrafish using both an anti-GFP antibody and FM1-43FX staining. Similarly, we found few GFP-positive or FM1-43FX-positive HCs in the neuromasts (Figure 7D–7H). Together these results suggest that loss of function of the *slc4a2b* gene disrupts HC development and function.

### *slc4a2b* mRNA injection can rescue the phenotype induced by *slc4a2b* gene deficiency

**Figure 8:**
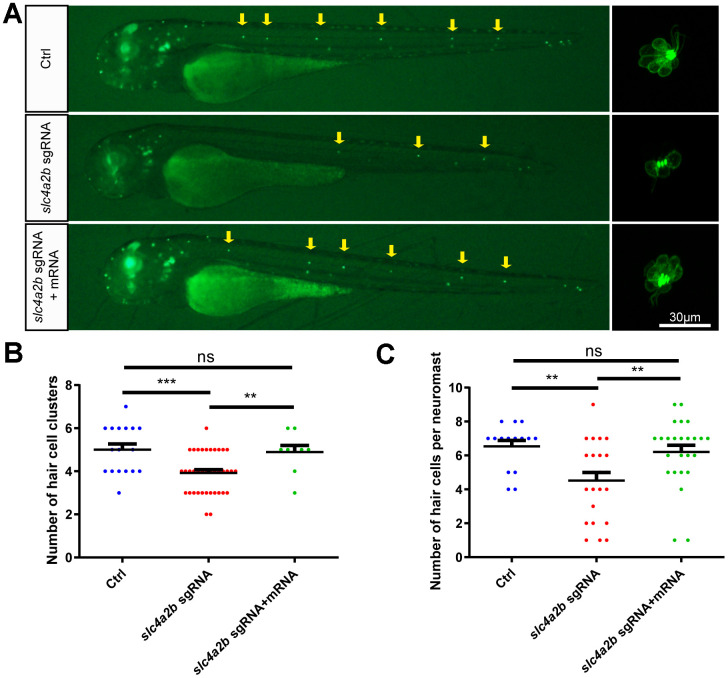
***slc4a2b* mRNA injection can rescue the phenotype induced by *slc4a2b* gene deficiency.** The *Tg(Brn3c:mGFP);slc4a2b*^mut^ zebrafish that were injected with Cas9 mRNA and *slc4a2b* sgRNA had fewer HC clusters (**A**, **B**) and fewer HCs in each posterior lateral line neuromast (**A**, **C**) compared to controls. However, *slc4a2b* mRNA injection could rescue the phenotype caused by *slc4a2b* mutation. *** *P* < 0.001; ** *P* < 0.01; ns, no significance.

To further verify that the phenotype of *slc4a2b* mutant zebrafish was due to the loss of function of *slc4a2b* rather than to nonspecific effects, we performed a rescue experiment by injecting *slc4a2b* mRNA together with the *slc4a2b* sgRNA and Cas9 mRNA into 1-cell-stage *Tg(Brn3c:mGFP)* zebrafish embryos. Zebrafish injected with *slc4a2b* mRNA had significantly higher *slc4a2b* expression level (Supplementary Figure 2), and had more HC clusters and HCs in the posterior lateral line in comparison with those without *slc4a2b* mRNA injection (Figure 8). This significant difference between the two groups provided strong evidence that *slc4a2b* gene mutation affected the development of posterior lateral line HCs, and because *slc4a2b* mRNA injection could rescue this deficiency, this suggests that *slc4a2b* is required for HC development in zebrafish.

### Loss of function of the *slc4a2b* gene leads to HC apoptosis

**Figure 9:**
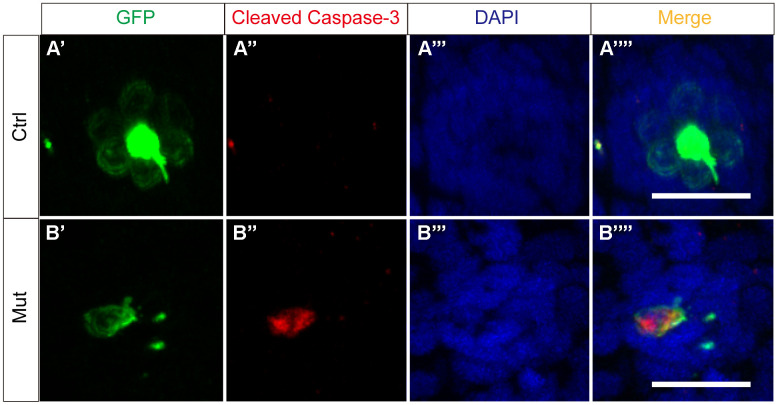
***slc4a2b* gene mutation leads to HC apoptosis.** The control zebrafish, *Tg(Brn3c:mGFP)* (**A**), and the mutant zebrafish, *Tg(Brn3c:mGFP);slc4a2b*^mut^ (**B**), were used for the apoptosis assay. Here, the anti-cleaved caspase-3 antibody was used to detect the apoptotic cells. Scale bar = 20 μm.

To determine if apoptosis is the primary mechanism through which *slc4a2b* gene defects lead to decreased numbers of HCs, we used an antibody against cleaved caspase-3, which is a marker of apoptosis. As shown in Figure 9, we observed GFP and cleaved caspase-3 double-positive cells in the posterior lateral line neuromasts of *Tg(Brn3c:mGFP);slc4a2b*^mut^ zebrafish indicating that loss of function of *slc4a2b* leads to HC apoptosis.

## DISCUSSION

Zebrafish is an excellent animal model owing to its optical transparency, high rate of reproduction, ectogenesis, and known genome sequence that has high homology with the human genome. Zebrafish also have many organs and systems that are similar to those of mammals and humans. For example, the zebrafish lateral line system, which consists of a number of neuromasts that enable the fish to perceive changes in its surroundings, consists of HCs, supporting cells and mantle cells. These HCs have similar morphologies and functions in the transmission of sensory information as mammalian HCs. The zebrafish lateral line neuromast is thus a very useful model to study the factors that affect HC development and function because this knowledge is likely to be applicable to our understanding of how dysfunction of inner ear HCs in mammals leads to hearing loss.

Recent decades have seen the identification of numerous genes that are responsible for hearing loss, and it is likely that there are still many deafness-related genes waiting to be discovered. Identifying such genes is a key step in a better understanding of the mechanisms of hearing loss and will form a fundamental basis for precise gene therapy in the future.

Ion homeostasis is essential for cell survival and function, and because HCs detect mechanical signals and transform them into electrical signals, ion homeostasis also plays an indispensable role in hearing. In HCs, some membrane-located transport proteins, for example, ion channels, transporters, and exchangers, play key roles, and dysfunction of these proteins leads to disruption of ion homeostasis and ultimately to cell death and subsequent hearing impairment.

The SLC protein family is made up of important solute carriers, and these proteins play vital roles in maintaining the physiology and function of numerous cell types. SLC4A2 is a sodium-independent anion exchanger of HCO_3_^−^ and Cl^−^, and it ensures that the pHi is maintained within a narrow range. The zebrafish *slc4a2b* gene is a member of the slc4 gene family, but its role in zebrafish development has not been reported until now.

First, we analyzed the evolutionary conservation and expression pattern of the zebrafish *slc4a2b* gene. The phyloP8way conservation score analysis and the WISH experiment showed that the *slc4a2b* gene is highly conserved and is mainly expressed in the caudal vein, otic vesicle, and neuromasts at different developmental stages. We next examined the role of *slc4a2b* in zebrafish lateral line neuromast HC development using morpholino-mediated gene knockdown and CRISPR/Cas9-mediated gene editing methods. Loss of function analysis showed that the zebrafish *slc4a2b* gene is required for HC development. Further, we showed that the phenotype induced by *slc4a2b* gene deficiency can be rescued by *slc4a2b*-specific mRNA injection, and this verified the role of the *slc4a2b* gene. Finally, we sought to explain how *slc4a2b* gene defects can lead to decreased HCs. Staining for the apoptosis marker cleaved caspase-3 showed that *slc4a2b* deficiency results in HC apoptosis in the posterior lateral line neuromast.

Apoptosis is one of the main types of cell death and is characterized by chromatin condensation and DNA degradation, and it can be divided into an extrinsic (or death receptor) and an intrinsic (or mitochondrial) pathway [19, 20]. Apoptosis is considered an important mechanism in the sensory HC death induced by aminoglycosides, cisplatin, and noise exposure. Several factors, including cysteinyl aspartate-specific proteinases (caspases) [21–23], B-cell lymphoma-2 family members [24–26], reactive oxygen species [27–29], c-jun NH2-terminal kinase pathway molecules [30–32], the p53 tumor suppressor gene [33–36], and some microRNAs [37–40], are known to play crucial roles in HC apoptosis. Caspase-3 activation is a key step and has been seen in HC apoptosis caused by aminoglycosides [41–44], cisplatin [34], and acoustic trauma [45–47]. Here, we demonstrate that *slc4a2b* gene deficiency can induce HC apoptosis mediated by caspase-3 activation in zebrafish.

Intracellular pH has a critical role in the maintenance of normal cell function and cell survival. pH regulation is important for hearing as mutation in H^+^-ATPases are known to cause hearing loss in human deafness patients with distal tubular acidosis [48]. And it has also previously been shown to be important in hair cells of zebrafish as raising fish mutating *gcm2*, a global pH regulator, or raising fish in altered pHs have both been shown to decrease hair cell number and reduce hair cell function [49, 50].

Taken together, these results indicate that the *slc4a2b* gene is required for HC development in zebrafish and that *slc4a2b* gene deficiency leads to increased HC apoptosis. Given that SLC4A2 is involved in pHi regulation and that *Slc4a2* deficiency alters pHi homeostasis in mice [9, 51, 52] and that disrupted pH homeostasis is an initiator of cell apoptosis [53, 54], we hypothesize that the HC apoptosis induced by the *slc4a2b* gene defect is due to changes in intracellular physiological parameters such as pHi, but this needs to be confirmed in future studies.

Zebrafish share approximately 70% of their genes with humans, and more than 80% of known human disease-related genes have a zebrafish counterpart [55]. Based on the evidence presented here, we propose that the human *SLC4A2* gene, the homologue of the zebrafish *slc4a2b* gene, is a potential deafness gene, and this should be confirmed in clinical studies.

## MATERIALS AND METHODS

### Zebrafish strains and maintenance

Two zebrafish lines were used in this study, the wild-type AB line and the transgenic line *Tg(Brn3c:mGFP)*. In the latter, membrane-localized green fluorescent protein (GFP) is expressed specifically in the HCs [16]. All of the zebrafish were raised at 28.5°C.

### The phyloP8way conservation score analysis

The phyloP8way conservation score file for zebrafish (genome reference version: danRer7) was downloaded from the UCSC Browser (http://hgdownload.soe.ucsc.edu/goldenPath/danRer7/phyloP8way/vertebrate.phyloP8way.bw), where the 8 species used for the conservation score calculation are *Homo sapiens* (hg19), *Mus musculus* (mm9), *Xenopus tropicalis* (xenTro2), *Tetraodon nigroviridis* (tetNig2), *Takifugu rubripes* (fr2), *Gasterosteus aculeatus* (gasAcu1), *Oryzias latipes* (oryLat2), and *Danio rerio* (danRer7). The corresponding gene annotation gtf file was downloaded from Ensembl (ftp://ftp.ensembl.org/pub/release-74/gtf/danio_rerio/Danio_rerio.Zv9.74.gtf.gz). The gtf annotation file was converted into bed format using a homemade python script. The bigWigAverageOverBed software from the UCSC browser was used to calculate the average phyloP8way conservation score for each protein-coding gene (only considering the coding sequence regions). The phyloP8way conservation score density plot was drawn using an R script.

### Whole-mount *in situ* hybridization

The whole-mount *in situ* hybridization (WISH) was performed according to standard procedures. First, a part of coding sequence of *slc4a2b* gene, amplified from zebrafish cDNA using the primers (5’-CAG CAT GGA TGA AGT GAC GG-3’ and 5’-CAG AAC CCT TGA CCA GCA TG-3’), was subcloned into the pGEM-T Easy vector, and then a gene-specific digoxigenin-labeled RNA probe was transcribed *in vitro* using the DIG RNA Labeling Kit (SP6&.T7) (Roche, #11175025910) following the manufacturer’s instructions. The pre-fixed embryos were incubated with the probe overnight at 4°C. Second, an alkaline phosphatase (AP)-conjugated antibody against digoxigenin (Roche, #11093274910) was used to detect the digoxigenin-labeled RNA probe. Finally, the AP-substrate NBT/BCIP solution (Roche, #11681451001) was added to the reaction system, and the development of color allowed the expression of the gene of interest to be visualized.

### Morpholino-mediated gene knockdown of *slc4a2b* in zebrafish

The oligo sequence of the *slc4a2b* gene-specific morpholino was 5’-TCA GGA CAC TGT GAA CCC GCT GAA C-3’, and it was obtained from Gene Tools, LLC. In this study, 2–3 nL of 0.3 mM morpholino oligo was microinjected into the embryos at the 1-cell stage. The morpholino blocks the translation of the Slc4a2b protein when the oligo binds to the *slc4a2b* mRNA.

### CRISPR/Cas9-mediated gene editing of *slc4a2b* in zebrafish

To generate the *slc4a2b* gene mutant zebrafish, as described in our previous work [56], 2–3 nL of a solution containing specific single guide RNA (sgRNA), which targeted exon 2 of the *slc4a2b* gene, and Cas9 mRNA was microinjected into each zebrafish embryo at the 1–2 cell stage. For the sgRNA synthesis, a forward primer (5’- TAA TAC GAC TCA CTA TAA GAC CAA CTC ACC AAC GCT GGT TTT AGA GCT AGA AAT AGC-3’), which contained a T7 promoter region and a *slc4a2b* gene-targeting region, and a universal reverse primer (5’-AAA AAA AGC ACC GAC TCG GTG CCA C-3’) were used in the PCR reaction with pT7 plasmid (Supplementary Figure 1) as the template to obtain the sgDNA that was then transcribed into sgRNA *in vitro* using the MAXIscript® Kit (Ambion, #AM1308). For the Cas9 mRNA synthesis, a linearized pXT7-Cas9 plasmid was used as the template for transcription into Cas9 mRNA *in vitro* using the mMESSAGE mMACHINE® Kit (Ambion, #AM1340).

### mRNA rescue experiment

In the rescue experiment, 2–3 nL of 50 ng/μL *slc4a2b* mRNA was co-injected into the embryos with the *slc4a2b* sgRNA. The mRNA was transcribed *in vitro* using the mMESSAGE mMACHINE® Kit (Ambion, #AM1340). Briefly, approximately 3700 bp DNA fragments were synthesized by PCR using the primers *slc4a2b*-mRNA-EcoRI-F (5’-CGG AAT TCC GTG AGG TTA TGC TGC CCG TAA-3’) and *slc4a2b*-mRNA- XbaI-R (5’-GCT CTA GAG CTG ATA GCA GCT CAA ACG CTC-3’). The DNA fragments were subcloned into the pCS2+ vector, and the recombinant plasmid was linearized using the restriction endonuclease Not I and then transcribed into mRNA *in vitro*.

### FM1-43FX staining

The vital dye FM1-43FX (Molecular Probe, #F35355) was used to specifically label functional HCs in the neuromasts. First, the live zebrafish were anesthetized using MS-222 and then immersed into the labeling solution for 45 s at room temperature in the dark. Afterwards, the fish were rinsed three times using the egg water and then fixed for 2 hours at room temperature in 4% paraformaldehyde. After washing three times with PBS-T (0.1% Triton X-100 in PBS), 4',6-diamidino-2-phenylindole (DAPI) was added to label the nuclei.

### Immunofluorescence

For immunofluorescence, the fish were anesthetized and then fixed using 4% paraformaldehyde. After washing three times with PBS-T, the fish were incubated in the antigen retrieval solution (Beyotime Biotechnology, China, #P0088) for 15 min at 98°C. Nonspecific binding was then blocked with 10% donkey serum in PBS-T. Next, specific primary antibodies against GFP (Abcam, #ab13970) and cleaved caspase-3 (CST, #9664) were added, and secondary antibodies were used to detect the primary antibodies.

### Statistical analysis

All data were analyzed using GraphPad Prism 8.0.2. Two-tailed, unpaired Student’s t-tests were used to determine statistical significance when comparing two groups. A value of *P* < 0.05 was considered statistically significant. All data are presented as means with SEM, and all experiments were repeated at least three times.

### Ethical approval

All animal procedures were performed according to protocols approved by the Animal Care and Use Committee of Southeast University and Nantong University and were consistent with the National Institutes of Health Guide for the Care and Use of Laboratory Animals. All efforts were made to minimize the number of animals used and to prevent their suffering.

## Supplementary Material

Supplementary Figures
