# Supplementary material for: The slc4a2b gene is required for hair cell development in zebrafish
Source: Aging (Albany NY). 2020 Oct 12;12(19):18804–21. doi: 10.18632/aging.103840 (PMC7732325; doi:10.18632/aging.103840)
Supplement: Supplementary Figures [file aging-12-103840-s001..pdf]

SUPPLEMENTARY FIGURES

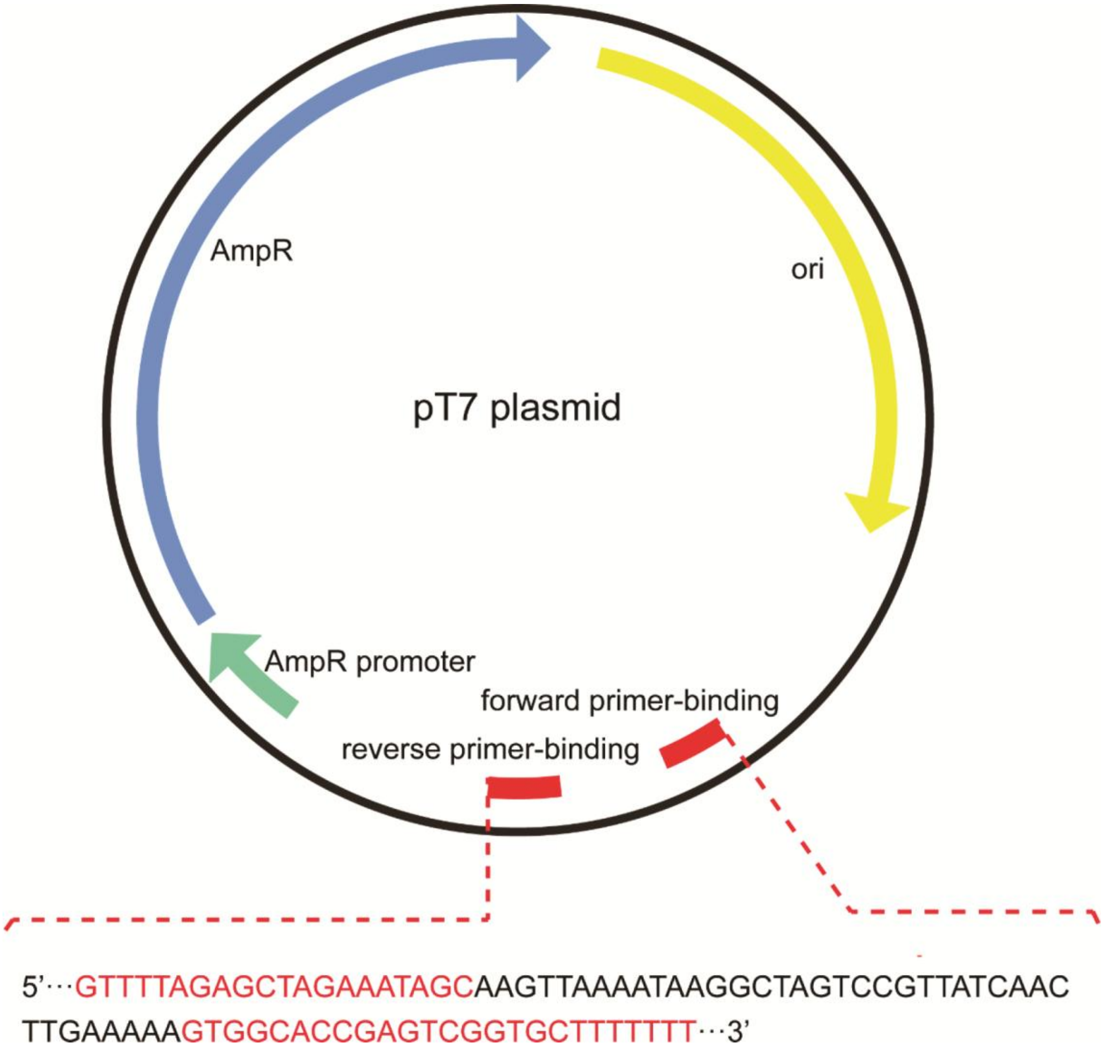

Supplementary Figure 1. The template plasmid used for sgRNA synthesis.

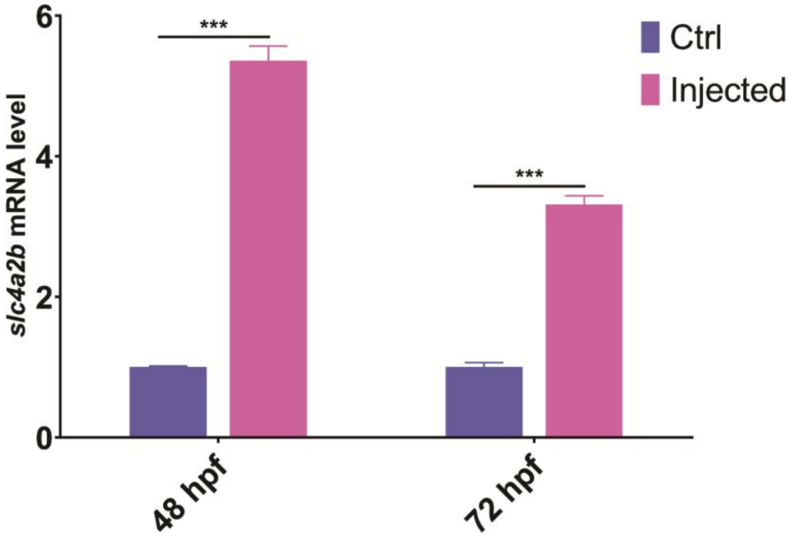

Supplementary Figure 2. The *slc4a2b* mRNA injection can significantly increase the *slc4a2b* expression level. After injecting with *slc4a2b* mRNA at 1-cell stage, zebrafish have significantly higher *slc4a2b* expression level at both 48 hpf and 72 hpf compared to the controls. Here, quantitative real-time PCR was used to analyze the *slc4a2b* mRNA expression level. The primers used were 5'-GTC CGA GTT TCA CAG AGG GAG-3' and 5'-GGG AGG ACT GAC GGT GAT AC-3'.
